# The Late Dr. Addison Niles

**Published:** 1875-05-01

**Authors:** 


### THE LATE DR. ADDISON NILES.

DIED, at his residence in Quincy, Ill., April 5th, 1875, Dr. ADDISON NILES, aged 63.

Dr. Niles was born at Plattsburgh, Steuben county, N. Y., April 26th, 1812. He commenced the study of medicine while young, though exactly at what date, cannot now be remembered; but he studied and practiced with his father, Dr. Noah Niles, for some years before graduating. He first attended lectures in 1833–4, at the medical college of Geneva, N.Y., where he graduated in 1835, after attending two courses. He subsequently attended another course at Geneva, in 1845–6; and during the following winter (1846–7), we find him at Jefferson Medical College, Philadelphia. He resided in Plattsburgh, Avoca, and Bath, of Steuben county, N.Y., until 1855, when he removed to Lockport, in Niagara county. Finally, in November, 1861, he removed to Quincy, Ill., at the solicitation of friends residing in this city, where he resided, engaged in a busy practice until his death, which occurred on the evening of April 5th, 1875.

Dr. Niles may be said to have lived solely in and for his profession, to which he was thoroughly and devotedly attached; few — of his medical friends, at least—ever heard him discourse upon any other subject. He was an indefatigable laborer in the cause of science, and possessed probably the largest and best selected medical library in this section. He had been, in former years, a contributor to some of the medical journals, and there is little doubt that he left a large amount of manuscript in the form of notes, essays, etc., which being founded on the experience of over forty years of practice, and containing in all probability, many peculiar and original ideas, cannot but be of interest; and as the doctor's son, Mr. John W. Niles is engaged in the study of medicine, it is to be hoped that these valuable papers may not be allowed to rest in oblivion.

In his intercourse with his professional brethren, Dr. Niles was somewhat reserved; though courteous to all, he cultivated but few friends or intimate acquaintances, but he was highly respected and admired by those who knew him. To his patients, and especially to those of the poorer class, among whom he had many, he was ever kind and tender; the numbers that filled St. Peter's church at the funeral service, would alone suffice to tell in what esteem he was held by those who attended the last sad rites.

Of his last illness, but little is known, for with his habitual reserve he consulted none, and refused all aid; when his family, alarmed by his condition, requested counsel, those who were called, arrived but to find him on his dying bed. An autopsy held the following morning, revealed extensive organic lesions, that had probably existed for many years; a full account of this will be given by Dr. M. Rooney, in a report to the Medico-Pathological Society.

The loss occasioned by the death of Dr. Niles, will long be felt, and more especially by the Fellows of the Medico-Pathological Society, of which he was one of the founders, and in which he was an ardent and enthusiastic worker. May his memory serve to stimulate his surviving Fellows, to renewed and never-failing exertion in the path which he so lately trod, and in the labors from which he is now at rest.

---

At a special meeting of the Medico-Pathological Society of Quincy, Illinois, held this day, the following preamble and resolutions were unanimously adopted:

"WHEREAS, We have learned with profound sorrow, of the death of DR. ADDISON NILES, one of the original Fellows, and a past President of this Society; therefore

"*Resolved*, That in his death, we mourn, in common with the entire medical profession of the city and county, the loss of one whose untiring devotion to science might well be held up as an example to his Fellows; whose unbending integrity commanded our fullest respect, and whose unfailing courtesy in all his professional relations, had won our highest esteem.

"*Resolved*, That we tender to the family of the deceased, that sympathy which comes from hearts suffering with them a common bereavement, and cherishing with them the honored name of him who has gone before."

It was ordered, that a copy of the above resolutions be sent to the family of the deceased, and that they be published in the Quincy *Whig* and *Herald*, and in the Chicago MEDICAL EXAMINER; also, that they be inscribed on the memorial pages of the Society's records, with such facts as may serve as a synopsis of the medical history of the deceased.

L. H. COHEN, M.D.,
*Secretary*.

QUINCY, Ill., April 6th, 1875.

